# Cellular signaling pathways in the nervous system activated by various mechanical and electromagnetic stimuli

**DOI:** 10.3389/fnmol.2024.1427070

**Published:** 2024-10-04

**Authors:** Youngjae Ryu, Aboubacar Wague, Xuhui Liu, Brian T. Feeley, Adam R. Ferguson, Kazuhito Morioka

**Affiliations:** ^1^Department of Neurobiology, University of Massachusetts Chan Medical School, Worcester, MA, United States; ^2^Department of Veterans Affairs, San Francisco Veterans Affairs Medical Center, San Francisco, CA, United States; ^3^Department of Orthopaedic Surgery, University of California, San Francisco, San Francisco, CA, United States; ^4^Department of Neurological Surgery, University of California, San Francisco, San Francisco, CA, United States; ^5^Brain and Spinal Injury Center, Zuckerberg San Francisco General Hospital and Trauma Center, San Francisco, CA, United States; ^6^San Francisco Veterans Affairs Healthcare System, San Francisco, CA, United States; ^7^Zuckerberg San Francisco General Hospital and Trauma CenterOrthopaedic Trauma Institute, , San Francisco, CA, United States

**Keywords:** mechanotransduction, nervous system, mechanical stress, cellular signaling, mechanosensor

## Abstract

Mechanical stimuli, such as stretch, shear stress, or compression, activate a range of biomolecular responses through cellular mechanotransduction. In the nervous system, studies on mechanical stress have highlighted key pathophysiological mechanisms underlying traumatic injury and neurodegenerative diseases. However, the biomolecular pathways triggered by mechanical stimuli in the nervous system has not been fully explored, especially compared to other body systems. This gap in knowledge may be due to the wide variety of methods and definitions used in research. Additionally, as mechanical stimulation techniques such as ultrasound and electromagnetic stimulation are increasingly utilized in psychological and neurorehabilitation treatments, it is vital to understand the underlying biological mechanisms in order to develop accurate pathophysiological models and enhance therapeutic interventions. This review aims to summarize the cellular signaling pathways activated by various mechanical and electromagnetic stimuli with a particular focus on the mammalian nervous system. Furthermore, we briefly discuss potential cellular mechanosensors involved in these processes.

## Introduction

The mammalian brain and spinal cord are viscoelastic tissues composed of heterogeneous cell populations, including neurons, astrocytes, oligodendrocytes, vascular endothelial, and microglia (Goriely et al., 2015). The stiffness of human and rodent brain tissue, with a shear modulus of approximately 1 kPa, is notably softer than that of other organ tissues (Elkin et al., 2010; Budday et al., 2017). Thus, nervous tissue is particularly sensitive to mechanical stimulation and easily susceptible to stress-induced cellular and axonal deformation (Reiter et al., 2023). However, it is well-established that mechanical stimuli influence cellular development, proliferation, and function, depending on the type and intensity of the stimuli. Therefore, the impact of mechanical stimuli on the brain and spinal cord must be considered in different contexts and applications.

Studies on traumatic injury and neurodegenerative disorders provide great insight into how mechanobiological forces influence the central nervous system (CNS). Focal traumatic injuries often result from the tension, compression and tearing of nervous tissue due to external stress (Blennow et al., 2012). Additionally, shear stress can occur when the brain experiences longitudinal tension and compression when acceleration is misaligned with the brain’s axis (Proces et al., 2022). These types of injuries serve as prime examples of how high levels of external stress impact the CNS. In neurodegenerative cases of such as Alzheimer disease or gliosis, an overall softening of nervous tissue is commonly observed, indicating a decrease in tissue stiffness (Hall et al., 2021). Beyond pathological conditions, mechanical forces generated during activities like sports or running can influence the brain and spinal cord tissues through flexion, extension, compression and shearing.

Numerous studies have investigated the effect of mechanical stress on the CNS through various methods of mechanical stimulation. However, these experiments have mainly focused on tissue-level effects and their macroscopic negative outcomes, such as diffuse axonal injury. Interestingly, mechanical stimulation can also have positive effects on the CNS. For instance, mechanical stretching promotes neurite outgrowth (Higgins et al., 2013) and induces changes in extracellular matrix stiffness, both known to regulate adult neurogenesis in the brain (Kjell et al., 2020). Therefore, the potential of mechanical stimulation to promote neural regeneration warrants consideration. To establish a standard for mechanical stimulation experiments and gain a more comprehensive understanding of its impact, it is essential to first evaluate the existing knowledge on the cellular and molecular outcomes of such stimuli.

Mechanical stimuli generated by dynamic movements, physical forces, and surrounding tissue are likely to influence the functions, maintenance and fates of neuronal and glial cells (Pillai and Franze, 2024). Additionally, these stimuli may share critical features in mechanotransduction. To fully understand the effects of mechanical stimuli on the CNS, it is necessary to investigate the key cellular signaling pathways involved. Several insightful reviews have examined the relationship between mechanical stimuli and the nervous system (Goriely et al., 2015; Proces et al., 2022; Motz et al., 2021; Rocha et al., 2022; Keating and Cullen, 2021). These reviews have explored mechanosensation, potential sensors, relevant protein channels (Proces et al., 2022; Hall et al., 2021; Higgins et al., 2013; Kjell et al., 2020), and the impact of various subtypes of mechanical stimuli in the context of CNS injuries and pathological conditions (Goriely et al., 2015; Rocha et al., 2022; Keating and Cullen, 2021). However, unlike other organs and cell types, few reviews address the causal effects of mechanical stimuli on neurons and glial cells, and the subsequent activation of cellular signaling pathways.

To summarize cellular pathways triggered by mechanical stimuli in the CNS, we first selected types of stimulation commonly studied in neurotraumatic research, such as stretching, compression and shear stress. We have also included fluid shear stress, which is known to be generated when external forces are applied to the brain or spinal cord (Keating and Cullen, 2021). Although secondary, mixed cascades, such as inflammation, inevitably occur after *in vivo* application of stress, leading to what is often termed ‘mechanical injury’, these studies still provided valuable insight. We prioritized studies that explicitly targeted mechanobiogical approaches or applied *in vitro* techniques specific to certain cell types.

In addition to mechanical stimulation, this review also encompasses cellular signaling pathways in the CNS driven by ultrasound and electromagnetic stimulation. Ultrasound is a mechanical pressure wave, while electromagnetic stimulation, such as transcranial magnetic stimulation (TMS), mediates biomolecular transduction through electromagnetic forces (Adey, 1993). Emerging studies suggest that non-invasive ultrasound and electromagnetic stimulation to the brain can induce therapeutic effects on psychological and neurodegenerative disorders (Nardone et al., 2014; George et al., 1999; Meng et al., 2021; Qiu et al., 2021). Therefore, it is valuable to consider how these external stimuli activate biomolecular pathways compared to classical mechanical stimuli.

In this review, we examine prominent mechanotransduction cellular signaling pathways, including MAPK, PKC, Akt, YAP/TAZ, and Rho-ROCK, which are activated by various types of mechanical and electromagnetic stimuli within the mammalian nervous system. These types of mechanical stimulation were selected for their practical interpretations and potential clinical implications. Additionally, we briefly discuss the mechanosensors and channels expressed on neurons and glial cells that are associated with the mechanotransduction of these stimuli.

### Mechanical and electromagnetic stimuli induced intracellular signaling pathways in the nervous system

#### MAPK signaling pathway

**Figure 1:**
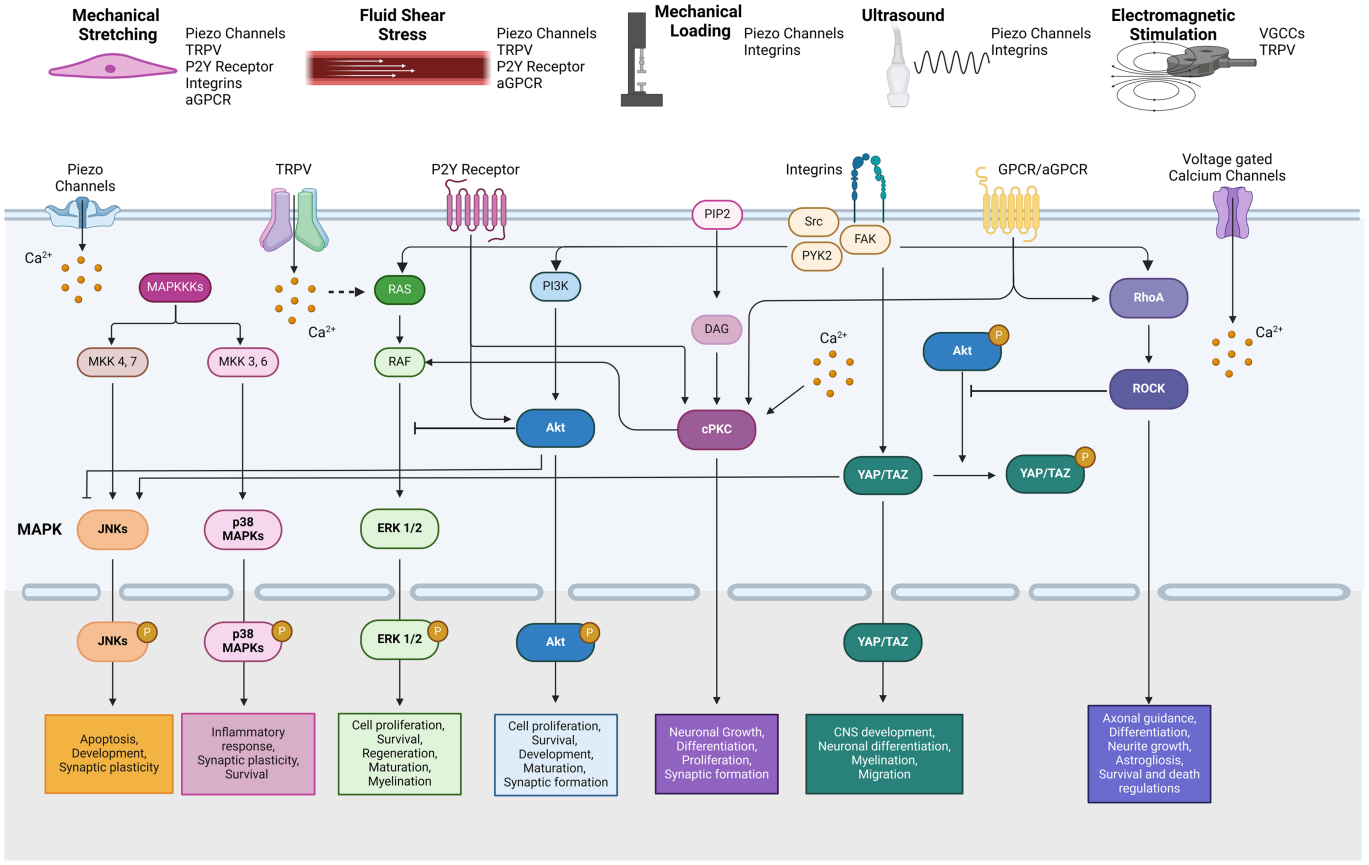
Types of mechanical stimulation and related mechanotransduction pathways by reported mechanosensors.

The mitogen-activated protein kinase (MAPK) pathway is one of the most well-known signal transduction cascades activated by various mechanical stimuli. MAPKs are categorized into three distinct types: extracellular signal-regulated kinases (ERKs), c-Jun N-terminal kinases (JNKs), and p38 MAPKs (Zhang and Liu, 2002). While the upstream signals within the MAPK network are interconnected, the activation of downstream MAPK family members is tightly regulated. These pathways have been studied in depth due to their significance in cell processes including, proliferation, differentiation, maintenance, and development in mammalian cells. In the nervous system, MAPK signaling has been implicated in neuronal development, axonal outgrowth, differentiation, maturation, and synaptic plasticity (Thomas and Huganir, 2004). In particular, JNKs, which are known to be activated by cellular stress, play key roles in regulating apoptosis and neuronal development (Coffey, 2014). Additionally, various cellular stresses such as oxidative stress, inflammatory cytokines or UV radiation, can trigger p38 MAPK activation, which regulates neuroinflammatory responses, survival and synaptic plasticity (Asih et al., 2020) (Figure 1).

Mechanical stretching has been shown to induce of phosphorylation of ERK1/2, JNKs, and p38 MAPKs across various cell types including cardiomyocytes, skeletal muscle cells, osteoblasts, and vascular endothelial cells (Hsu et al., 2010; Suzuma et al., 2002; Zou et al., 2004; Boppart et al., 2001; Kanno et al., 2007). The degree of MAPK activation correlates positively with both the percentage (Aikawa et al., 2002) and frequency (Hz) of stretch (Hsu et al., 2010). Studies indicate that mechanical stretching activates MAPK signaling in astrocytes, cortical neurons, and oligodendroglial cell lines (Neary et al., 2003; Chierto et al., 2019; Ralay et al., 2011; Bhowmick et al., 2019). For example, in primary cultured rat astrocytes, 30% elongation under mechanical stretch initially elevates phospho-ERK levels, while prolonged stretching up 24 h is needed to increase activation of JNKs (Ralay et al., 2011). However, levels of activated p38 MAPKs were not significantly impacted by stretch. The stretch-induced ERK activation in primary rat astrocytes is hypothesized to be calcium-dependent and mediated by ATP-release in response to strain (Neary et al., 2003). These studies suggest that MAPK activation is related to the cellular mechanism of astrogliosis formation and inflammatory responses after mechanical stress.

In biaxially stretched cortical neurons, 30% stretch resulted in increased phosphorylation of ERK 1/2 and JNKs, but not p38 MAPKs (Bhowmick et al., 2019). These findings imply that ERK and JNK activations significantly regulate apoptotic neuronal cell death. In contrast, a study using the oligodendroglial cell line 158 N found that a 30% stretch stress did not significantly alter phosphorylated ERK 1/2 and JNKs levels, but reduced the levels of phosphorylated p38 MAPKs (Chierto et al., 2019). This tensile strain led to increased reactive oxygen species and changes in myelination protein expression. However, an *ex vivo* stretch test on mouse cerebellar tissue showed ERK 1/2 activation, while activation of p38 MAPKs was not observed. Additionally, in the retinal tissue layer, positive ERK 1/2 immunosignals increased following 20% stretch stress (Lindqvist et al., 2010).

Fluid shear stress has also been shown to induce phosphorylation of JNKs, ERKs, and p38 MAPKs in *in vitro* systems using vascular endothelial cells, osteosarcoma cell lines, and osteoblasts (Kadohama et al., 2006; Mengistu et al., 2011; Kapur et al., 2003). The extent of phosphorylation is influenced by both the duration (minutes) and the magnitude of stress (dyn/cm^2^ or Pa) applied. However, it has also been reported that laminar fluid shear stress inhibits TNF-*α* and IL-1-mediated activation of JNKs in human umbilical vein endothelial cells (Surapisitchat et al., 2001). Although fluid shear stress influences N-methyl-D-aspartic acid (NMDA) receptor-mediated calcium influx in primary cultured rat astrocytes (Maneshi et al., 2017), there have been no reports on the direct application of fluid shear stress to other glial or neuronal cells *in vitro* in relation to MAPK signaling.

Mechanical pressure can generate compression and shear strain in the tissue. It has been reported that *in vitro* compression and shear strain can activate ERKs, JNKs, and p38 MAPKs in chondrocytes as well as osteoblasts (Song et al., 2016; Fitzgerald et al., 2008). However, there is a paucity of similar studies using neural cells. *In vivo* models mild traumatic brain injury can be viewed as mechanical stimuli models that incorporate both compression and shear stress (Keating and Cullen, 2021). In these models, significant phosphorylation of ERKs and p38 MAPKs have been observed in mouse brain tissue (Bachstetter et al., 2013; Zhao et al., 2012), while JNK activation was not detected (Mori et al., 2002). Additionally, chronic compression of the dorsal root ganglion (DRG) *in vivo* resulted in activation of ERKs and p38 MAPKs in neurons and microglia (Han et al., 2012; Qu et al., 2016). These findings suggest that mechanical compression stimuli generally facilitate the activation of ERKs and p38 MAPKs, which regulate neuronal apoptosis and inflammatory responses, such as cytokines release. Although secondary inflammatory responses following *in vivo* mechanical stress may contribute to these effects, the involvement of MAPK cascades in neuroprotection is suggested.

Ultrasound can generate mechanical pressure waves that are typically applied both *in vitro* and *in vivo* at frequencies in the MHz range. In the medical setting, ultrasound is conducted at a frequency range between 1 to 15 MHz, while therapeutic applications typically utilize frequencies of around 1 MHz or lower (Tufail et al., 2011). Ultrasound can be delivered through various tissues as pulsed or continuous waves. Its physiological effects on cells and tissues have been attributed to a combination of heat generation, micro-vibrations, microstreaming, and shear stress (VanBavel, 2007; Xin et al., 2016). However, low-intensity pulsed ultrasound (LIPUS), which operates at intensities below 3 W/cm^2^, has minimal thermal effects and is primarily used as a therapeutic intervention (Jiang et al., 2019).

In mammalian osteoblast and synovial cells, LIPUS at frequencies between 1.5 and 3 MHz has been shown to upregulate phosphorylation levels of ERKs, JNKs, and p38 MAPKs (Chiu et al., 2008; Sato et al., 2014). In contrast, LIPUS stimulation in dental pulp stem cells led to increased activation of ERKs and JNKs, but not p38 MAPKs (Gao et al., 2017). In rodent 3 T3-L1, MC3T3-E1, and ST2 cells, LIPUS application at 1.5 MHz induced phosphorylation of ERK 1/2 only, without affecting JNKs and p38 MAPKs (Kusuyama et al., 2014). In addition, Louw et al. found that phosphorylated ERK levels did not change with ultrasound frequencies above 2 MHz in chondrocytes (Louw et al., 2013). However, the phosphorylation of ERKs was did not significantly change in rat astrocytes treated with LIPUS at 1 MHz frequency (Liu et al., 2017). Similarly, *in vivo* ultrasound application at 0.5 MHz did not promote the phosphorylation of JNKs or p44/42 MAPK in rat brain tissue (Jalali et al., 2010) although the applied frequency ranges in these reports are generally lower than those used in other system.

Electromagnetic stimulation also alters the cellular activation and function in the nervous system (Siebner et al., 2022). Theoretically, an electromagnetic field induces the vibration of free ions on the cellular membrane (Panagopoulos et al., 2002), modulating ion influx and membrane potential due to microelectronic activities. It has been suggested that electromagnetic stimuli, such as weak electromagnetic fields (EMF), can activate the MAPK pathway (Blank and Goodman, 2009). Extremely low-frequency EMF in the range of 0–100 Hz are commonly used and are considered to have negligible thermal effects at the cellular level (Adey, 1993). A study using low frequency electromagnetic stimulation of 50 Hz on various cell lines has shown an increase in the phosphorylation of ERK 1/2 and p38 MAPKs, but did not promote the phosphorylation of JNKs (Kapri-Pardes et al., 2017).

Focusing on the nervous system’s response to EMF, *in vitro* use of a repetitive transcranial magnetic stimulation (TMS) system at 10 Hz on rat neural stem cell cultures has demonstrated activation of ERK 1/2 and p38 MAPKs, while JNKs were not significantly affected (Cui et al., 2019). This suggests that the TMS-activated MAPK pathway may enhance the proliferation and differentiation of neural stem cells. Similarly, magnetic stimulation applied to mouse neuronal cells and oligodendrocyte precursor cell line CG4 resulted in a significant increase in the ERK 1/2 phosphorylation (Ma et al., 2013; Dolgova et al., 2021). In the mouse hippocampal neuronal cell line HT22, EMS at 10 Hz and 1.75 T intensity increased the levels of phosphorylated ERK 1/2 and JNKs, but not p38 MAPKs (Kim et al., 2023). Also, in the rat Schwann cells, 50 Hz magnetic stimulation at 0.1 T intensity transiently increased phospho-ERK 1/2 levels (Colciago et al., 2015). Overall, these studies indicate that activation of MAPKs by electromagnetic stimulation enhances synaptic plasticity, attenuates of cell death and promotes remyelination *in vitro*. Furthermore, *in vitro* exposure of electromagnetic pulses to rat microglia resulted in p38 MAPKs phosphorylation, but did not affect ERKs and JNKs (Yang et al., 2016). *In vivo*, repetitive TMS at 15 Hz frequency and 1.26 T intensity led to elevated ERK 1/2 phosphorylation and increased neuronal proliferation in hippocampal tissue (Chen et al., 2015). Additionally, TMS at 15 Hz induced robust axonal elongation and MAP2K phosphorylation in cortical neurons (Boato et al., 2023). Collectively, both mechanical and electromagnetic stimuli typically enhance ERK phosphorylation, whereas the activation of JNK or p38 MAPK is inconsistent and varies depending on the type of stimulus and cell type (Table 1).

**Table 1:** Summarized results of mechanically or electromagnetically activated intracellular pathways from studies using the mammalian nervous system.

Stimulation types	Test systems	MAPK	PKC	Akt	YAP/TAZ
Mechanical Stretching	Cortical neuron 30% stretch (2.0 psi) (Bhowmick et al., 2019)	ERK↑, JNK↑, p38━ (Bhowmick et al., 2019)	cPKC↑ (Zhang et al., 1996)	Akt↑ (Wang et al., 2023)	
	Astrocyte 30% stretch (Ralay et al., 2011) 20% cyclic stretch (Ostrow et al., 2000) 8–54% stretch (Neary et al., 2005)	ERK↑, JNK↑, p38━ (Ralay et al., 2011)	cPKC↑ (Ostrow et al., 2000)	Akt↑ (Neary et al., 2005)	
	Oligodendrocyte 30% stretch (Chierto et al., 2019) 15% stretch (Shimizu et al., 2017)	ERK↑, JNK↑, P38↓ (Chierto et al., 2019)			Nuclear YAP/TAZ↑215
	Schwann cell 150% stretch (Poitelon et al., 2016)				Nuclear YAP/TAZ↑
	*Ex vivo* rodent brain tissue 30% stretch (Chierto et al., 2019)	ERK↑, p38━			
	*Ex vivo* rodent retina 20% stretch (Lindqvist et al., 2010)	ERK↑			
Fluid Shear Stress	Neuronal cell line 1 Pa laminar FSS (Ryu et al., 2020)		cPKC↑		
	Oligodendrocyte (Shimizu et al., 2017)				Nuclear YAP/TAZ↑
Mechanical Compression	*In vivo* rodent brain compression (Mori et al., 2002; Zhao et al., 2012; Padmaperuma et al., 1996; Noshita et al., 2002)	ERK↑, JNK━, p38↑ (Mori et al., 2002; Zhao et al., 2012)	cPKC↑ (Padmaperuma et al., 1996)	Akt↑ (Noshita et al., 2002)	
	*In vivo* rodent spinal cord compression (Chen et al., 2016; Xie et al., 2020): Astrocyte focused			Akt↑ (Chen et al., 2016)	Nuclear YAP/TAZ↑ (Xie et al., 2020)
	*In vivo* rodent DRG compression (Han et al., 2012; Qu et al., 2016)	ERK↑, p38↑ (Han et al., 2012; Qu et al., 2016)			
Ultrasound	Astrocyte LIPUS 1 MHz, 110 mW/cm^2^ (Liu et al., 2017)	ERK━		Akt↑	
	Microglia LIPUS 1 MHz, 528 mW/cm^2^ (Su et al., 2023)			Akt↓	
	*In vivo* brain tissue FUS 0.5 MHz (Jalali et al., 2010)	JNK↑		Akt↑	
Electromagnetic Stimulation	Cultured neural stem cell EMF 10 Hz, 500 mV/div, 5 ms/div (Cui et al., 2019) MS 20 Hz, 0.87–1.58 T (Zhao et al., 2019)	ERK↑, JNK━, p38↑ (Cui et al., 2019)		Akt↑ (Zhao et al., 2019)	
	Hippocampal neuron MS 1 Hz, 1.14–1.55 T (Ma et al., 2013) MS 1 or 10 Hz, 1.75 T (Kim et al., 2023)	ERK↑, JNK↑, p38━ (Kim et al., 2023)		Akt↑ (Ma et al., 2013; Kim et al., 2023)	
	Cerebellar neuron EMF 50 Hz, 1 mT (Yang et al., 2015)		cPKC↑		
	OPC cell line MS 40 Hz (Dolgova et al., 2021)	ERK↑		Akt↑	
	Microglia EMP 200 kV/m (Yang et al., 2016)	ERK━, JNK━, p38↑			
	Schwann cell MS 50 Hz, 0.1 T (Colciago et al., 2015)	ERK↑ (transient)		Akt↑ (transient)	Nuclear YAP/TAZ↑
	*In vivo* brain EMF EMF 50 Hz, 0.1 mT (Manikonda et al., 2007)		cPKC↑		
	*In vivo* WT brain TMS 15 Hz, 60 pulse/train, 1.26 T (Chen et al., 2015) 15 Hz, 75 pulse/train (Boato et al., 2023)	ERK↑ (Chen et al., 2015)		Akt━ (Boato et al., 2023)	
	*In vivo* model brain TMS BDNF potentiated, 5 Hz, 400 pulse/train (Wang et al., 2011) MCOA model, 20 Hz, 1.4 T (Luo et al., 2017)			Akt↑	

cPKC, conventional PKC; FSS, fluid shear stress; DRG, dorsal root ganglia; LIPUS, low-intensity pulsed ultrasound; FUS, focused ultrasound; MS, magnetic stimulation; TMS, transcranial magnetic stimulation; EMF, electromagnetic field; EMP, electromagnetic pulse; MCOA, middle cerebral artery occlusion; ↑, activation.

#### PKC signaling pathway

Protein kinase C (PKC) is activated by mechanical stress via gated channels and receptors including the NMDA receptor, TRPV4, and the purinergic P2Y_2_ receptor (Lea et al., 2002; Wang et al., 2005; Adapala et al., 2011). Activation of PKC typically involves the participation of phospholipase and diacylglycerol leading to a cascade of downstream cellular signaling, including involvement of the MAPK pathway. PKC is classified into several subgroups: conventional (calcium-dependent;*α*, *β*, and *γ*), novel (calcium-independent; *δ*, *ε*, *η* and *θ*), and atypical types (Parekh et al., 2000). The expression of PKC isozymes varies depending on the cell type and tissue, influencing their specific signaling pathways. For example, PKCγ is highly expressed in the nervous tissue, PKCδ is predominantly found in the soma of neurons, and PKCε is concentrated in the presynaptic terminals (Liu and Heckman, 1998). PKC activation regulates many functions in the CNS, including neurotransmitter release, neuronal growth, proliferation and differentiation, and synaptic plasticity (Huang, 1989) (Figure 1).

Mechanical stretch activates various PKC isoforms across different cell types, including conventional PKC in fibroblast and smooth muscle cells, PKCζ in vascular pericytes and PKCα/ε in endothelial cells (Suzuma et al., 2002; Cheng et al., 2001; Husse et al., 2007; Persson et al., 1995). In cortical neurons and astrocytes, mechanical stretch-induced PKC activation is linked to calcium influx via the NMDA receptor (Lea et al., 2002; Zhang et al., 1996; Ostrow and Sachs, 2005). *In vitro* studies suggest that mechanical stretching not only activates the NMDA receptor, but also modifies the α-amino-3-hydroxyl-5-methyl-4-isoxazolepropionate (AMPA) receptor leading to potentiated calcium influx in neurons (Spaethling et al., 2012; Goforth et al., 2004). This stretch-induced calcium influx is thought to directly trigger PKC activation. A study investigating the activation of PKCα through mechanical stretching found that this process led to the internalization of the GluR2 subunit of the AMPA receptor in rat cortical neurons (Bell et al., 2009). This suggests a connection between traumatic CNS injury and neuronal toxicity due to the loss of GluA2-lacking AMPA receptor (Ferguson et al., 2008).

*In vitro* studies on endothelial cells have shown that both conventional and novel types of PKC isoforms are activated by fluid shear stress (Ishida et al., 1997; Berk et al., 1995). Conventional PKC activity is trigged through direct calcium influx, while the calcium-independent PKCε isoform is also activated by fluid shear stress, both mediate ERK 1/2 phosphorylation (Traub et al., 1997). *In vitro* studies utilizing neuronal cell lines have also shown PKC activation and elevated intracellular calcium levels in response to fluid shear stress (Ryu et al., 2020). However, research on PKC activation in other neural cell types in response to shear stress remains limited.

Compressive stimulation has been shown to activate the PKCα pathway in chondrocytes and keratinocytes through integrin-associated mechanotransduction (Takei et al., 1997; Lee et al., 2002). In human nucleus pulposus cells, mechanical compression specifically activates the PKCε isoform (Sun et al., 2013). In the nervous system, *in vivo* studies using compressive methods have reported upregulation of conventional PKC isoforms in brain and spinal cord neurons, particularly those associated pain sensitivity (Padmaperuma et al., 1996; Mao et al., 1992). Although these studies closely resemble ‘mechanical injury’, and should be interpreted as reflecting a combination of various effects on the tissue, the majority of PKC activation is still primarily attributed to mechanical stimulation. These studies observed significant membrane translocation of PKC in ipsilateral compressed nervous tissue, linking PKC activation with mechanical stress. This activation could result from either mechanically induced calcium influx or as part of cellular injury response, such as apoptosis. Direct evidence from *in vitro* studies or specific effects of PKC activation in neurons or glial cells induced solely by compression has yet to be reported.

Studies exploring the relationship between LIPUS and PKC activation are sparse. However, a study by Lee et al. demonstrated that exposure to 1 MHz ultrasound significantly elevated intracellular calcium levels and enhanced calcium-independent PKCδ activation in a murine skeletal muscle cell line (Lee et al., 2014). While there are currently no reports specifically addressing ultrasound-induced PKC activation in the CNS, these findings suggest the potential of a similar PKC activation mechanism in neural tissues.

The effects of EMF on PKC activation have been explored in lymphocytic cell lines such as HL60 and NALM6 (Uckun et al., 1995; Nie and Henderson, 2003), indicating that PKCα is particularly activated among the isozymes of PKC. In rat cerebellar neurons exposed to 50 Hz EMF, increased phosphorylation of PKC was observed with enhanced neuronal GABA_A_ receptor-mediated currents (Yang et al., 2015). Similarly, *in vivo* exposure of rats to 50 Hz EMF resulted in increased PKC activity and intracellular calcium levels, likely due to NMDA receptor activation (Manikonda et al., 2007). Even direct mild electrical stimulation at 10 Hz activated PKC in the astroglia cells, leading to the release of nerve growth factor (Koyama et al., 1997). However, these studies did not differentiate between PKC subgroups. In contrast, high-frequency 2–10 GHz microwave exposure in rats demonstrated reduced PKC activation in the cerebral cortex with accompanied by aggregation of glial cell populations and morphological changes (Paulraj and Behari, 2006). Overall, PKC activation has been commonly linked with an increase in intracellular calcium levels, regardless of the type of stimulation, highlighting its potential role in cellular responses to electromagnetic fields (Table 1).

#### Akt signaling pathway

The Akt signaling pathway, also known as Protein Kinase B, plays a crucial role in cellular proliferation, maintenance, and survival, similar to the well-studied MAPK pathway. Akt activation is primarily mediated by the downstream effects of phosphoinositide 3-kinases (PI3K), which are transduced by various biological factors, including brain-derived neurotrophic factor (BDNF) and insulin-like growth factor 1 (IGF-1) (Brunet et al., 2001). The cascade of Akt phosphorylation leads to the activation of NF-κB and mTOR, key regulators of cellular processes. In the nervous system, Akt signaling is implicated in neuronal survival, development, maturation, axonal regeneration, and synapse formation (Sanchez-Alegria et al., 2018) (Figure 1).

Cyclic stretching has been shown to increase PI3K activity and Akt phosphorylation in osteoblasts, epidermal cells, endothelial cells, and vascular pericytes (Suzuma et al., 2002; Danciu et al., 2003; Kippenberger et al., 2005; Hu et al., 2013). Additionally, *in vivo* stretching has been found to enhance Akt activity in rat muscle tissue (Sakamoto et al., 2003). In primary cultured rat astrocytes, mechanical strain ranging from 8 to 54% significantly increased the levels of phosphorylated Akt (Neary et al., 2005). Neary et al. highlighted that activation of Akt in astrocytes is dependent on calcium influx triggered by mechanical stretching which is crucial for their survival and growth (Neary et al., 2005). Similarly, mechanical stretching of primary cultured mouse neurons also resulted in upregulation of Akt activity (Wang et al., 2023). Although detailed analyses on the mechanism behind Akt in response to mechanical stretching are limited, it is generally attributed to calcium influx and PI3K activation.

Akt activation has also been observed in endothelial cells and osteocytes subjected to fluid shear stress (Batra et al., 2014; Haga et al., 2003). However, there is no direct or indirect evidence of fluid shear stress affecting neurons or glial cells in relation to the Akt pathway. In regard to mechanical compressive stimulation, Akt activation has been observed in numerous cell types including osteoblasts, fibroblasts, and chondrocytes in rodent models (Song et al., 2016; Niehoff et al., 2008; Tanaka et al., 2018). Although *in vitro* studies on neuronal or glial cells are limited, *in vivo* studies have shown increased Akt phosphorylation in neurons and astrocytes within mouse brain and rat spinal cord tissues under compression (Chen et al., 2016; Noshita et al., 2002). These studies employed classical ‘mechanical injury’ techniques to induce stress, suggesting that the Akt signaling is activated in the ipsilateral tissues to prevent neuronal death. However, it remains uncertain whether Akt signaling would be activated under precise compression methods at the cellular level. Further studies are needed to explore this question.

LIPUS at 1.5 MHz has been shown to activate the PI3K-Akt signaling pathway in osteoblast (Tang et al., 2006).Similarly, LIPUS application at 110 mW/cm^2^ and 1 MHz significantly increased phosphorylated Akt in rat astrocytes and elevated BDNF levels (Liu et al., 2017). *In vivo* studies on rat brains exposed to LIPUS also demonstrated enhanced Akt activation which correlated with increased VEGF expression and paracellular permeability (Jalali et al., 2010). However, in rat microglia, PI3K-Akt signaling was reduced after LIPUS application at 528 mW/cm^2^ and 1 MHz, accompanied by decreased microglial activities and cytokine releases (Su et al., 2023). These conflicting findings suggest that activation of Akt by ultrasound varies depending on the specific cell type within the CNS.

Studies on electromagnetic stimulation have shown that exposure to 50 Hz EMF does not significantly alter Akt phosphorylation in several non-neural cell lines (Kapri-Pardes et al., 2017). However, rodent-cultured neuronal cells exposed to magnetic stimulation experienced increased phosphorylated Akt in neural stem/precursor cells, oligodendrocyte precursor cell lines, and hippocampal cell lines (Ma et al., 2013; Dolgova et al., 2021; Kim et al., 2023; Zhao et al., 2019). Furthermore, in rat Schwann cells, 50 Hz of electromagnetic exposure resulted in a transient increase in phosphorylated Akt levels (Colciago et al., 2015). In specific conditions, such as the rat brain ischemic model and BDNF-induction pathway, TMS has been shown to significantly increase the levels of phosphorylated Akt in brain tissue, along with BDNF and tyrosine kinase B (Luo et al., 2017; Wang et al., 2011). Conversely, *in vivo* TMS at 15 Hz in wild-type adult mice did not activate Akt signaling in cortical tissue (Boato et al., 2023). Similarly, *in vivo* exposure of rats to high-frequency microwaves (1–2 GHz) did not result in significant changes of Akt phosphorylation levels in brain tissue (Tan et al., 2021). These findings suggest that the effects of electromagnetic stimulation on Akt activation vary depending on cell type and experimental conditions, including whether the study is conducted *in vitro, in vivo,* or pre-conditioned models (Table 1).

#### YAP/TAZ signaling pathway

Yes-associated protein (YAP) and its coactivator PDZ-binding motif (TAZ) are critical downstream effectors of the Hippo signaling pathway (Piccolo et al., 2014). YAP/TAZ acts as a transcriptional regulator with essential roles in developing the CNS, including neuronal differentiation, cellular migration, and apoptosis. Moreover, YAP/TAZ dysregulation has been implicated in neurodegenerative disorders such as Huntington’s or Alzheimer’s diseases (Jin et al., 2020). Phosphorylation of YAP is mediated by the Hippo pathway, particularly through the action of MOB1 and LATS1/2. Once phosphorylated, YAP/TAZ is sequestered in the cytoplasm, blocking its translocation to the nucleus and inhibiting transcriptional activity. It should be noted that YAP is not detectable in mature neurons (Huang et al., 2016). The nuclear activity of YAP/TAZ is regulated by integrin activation, which is triggered by a range of mechanical stimuli, including matrix stiffness, stretching, and cell density (Piccolo et al., 2014) (Figure 1).

The nuclear translocation of YAP/TAZ induced by mechanical stretching has been extensively studied in *in vitro* systems using epithelial cells, various stem cell lineages, and endothelial cells (Aragona et al., 2013; Elbediwy et al., 2016; Panciera et al., 2017). In rat oligodendrocyte progenitor cells, a 15% stretch was sufficient to promote nuclear localization of YAP (Shimizu et al., 2017). Similarly, Schwann cells exhibited increased YAP/TAZ nuclear activity under 150% mechanical stretch (Poitelon et al., 2016). While the role of matrix stiffness-related in regulating YAP/TAZ activity has been highlighted in glial cells and the differentiation of neural stem cells (Rammensee et al., 2017; Marinval and Chew, 2021), studies specifically employing direct stretching systems have not been reported. Nonetheless, it can be hypothesized that mechanical stretching could trigger YAP/TAZ nuclear activity, potentially influencing processes like myelination and cell morphology (Hong et al., 2023). Similarly, fluid flow-mediated nuclear translocation of YAP has been documented in endothelial cells and various tumor cell types (Yu et al., 2021; Qin et al., 2019; Nakajima et al., 2017). In oligodendrocytes, YAP activity was regulated by shear stress generated by rotating culture flasks (Shibasaki et al., 2010). However, studies explicitly investigating YAP/TAZ translocation in glial cells beyond this finding are scare.

Mechanical compression applied to rodent osteocytes and chondrocytes has been shown to promote the nuclear translocation of YAP/TAZ (Zarka et al., 2021; Jing et al., 2020). In the CNS, rodent models have demonstrated that compressive stimulation increases YAP/TAZ nuclear translocation in astrocytes (Xie et al., 2020; Pu et al., 2023). This YAP activation was found to enhance astrocyte proliferation and is associated with the formation of astrogliosis. This process may play a role in regulating glial scar and promoting functional recovery following mechanical compression. Regarding ultrasound stimulation, LIPUS at 1.5 MHz for 20 min has been shown to increase YAP phosphorylation in human umbilical vein endothelial cells (Xu et al., 2018). However, in mouse C2C12 myoblast cell line, LIPUS at 3.6 MHz for 5 min reduced YAP phosphorylation and induced its nuclear translocation (Puts et al., 2018). These conflicting results, coupled with limited research on LIPUS effects on the nervous system, necessitate further investigation into the influence of ultrasound stimulation on YAP/TAZ activity.

There are several reports examining the effects of electromagnetic stimulation on YAP/TAZ activity. In human tendon cells, exposure to a 2 Hz EMF did not affect YAP/TAZ nuclear translocation (Pesqueira et al., 2017). Conversely, a static EMF of 1 mT applied to human dental pulp stem cells resulted in slight activation of YAP/TAZ nuclear activity (Zheng et al., 2018). Interestingly, when rat Schwann cells were exposed to a 50 Hz EMF, there was upregulation of cytoplasmic localization of YAP (Colciago et al., 2015). This shift was accompanied by enhanced proliferation and migration in EMF-exposed Schwann cells compared to the control group. However, studies specifically investigating the effects of EMF on YAP/TAZ in glial cells are lacking. From the current literature it can be reasonably assumed that the impact of electromagnetic stimulation on YAP/TAZ in the nervous system may be rather limited (Table 1).

#### Rho-ROCK signaling pathway

The Rho GTPase family, which includes Rho, Rac, and Cdc42, plays an essential role in common cellular processes including growth, migration, morphology, cell cycle regulation, and cytoskeletal structure remodeling (Etienne-Manneville and Hall, 2002). Among these, RhoA and its downstream effector, Rho-associated coiled-coil containing protein kinase 1 (ROCK), mediates mechanical signals through interactions between GPCRs and integrins (Buchsbaum, 2007). In the nervous system, the Rho-ROCK signaling pathway has been extensively studied for its role in promoting axonal guidance, neurite outgrowth, neuronal differentiation, and the regulation of astrocyte morphology astrogliosis (John et al., 2004; Stankiewicz and Linseman, 2014). Numerous studies have demonstrated that changes in the stiffness of the extracellular matrix (ECM) can activate the Rho-ROCK signaling pathway, which in turn regulates a range of cellular responses to these external mechanical cues (Zhang et al., 2017; Huang et al., 2012; Huang and Ingber, 2005).

Research using rodent cardiomyocytes and pluripotent stem cells has demonstrated that mechanical stretch activates the Rho-ROCK signaling pathway (Torsoni et al., 2005; Teramura et al., 2012). Similarly, fluid shear stress in mammalian endothelial cells induces Rho translocation to the cytoplasmic membrane, activating the Rho-ROCK pathway and its downstream effectors (Lin et al., 2003). In fibroblast, this pathway also mediates cellular stiffening in response to shear stress (Lee et al., 2006). Additionally, LIPUS has been shown to trigger the Rho-MAPK pathway in mouse phagocytes and mesenchymal stem/progenitor cells (Kusuyama et al., 2014; Zhou et al., 2008), while 50 Hz EMF promotes Rho-ROCK activation in human mesenchymal stem cells (Zhang et al., 2018). However, studies have yet to explore the effects of direct mechanical stress on Rho-ROCK signaling in the nervous system both *in vitro* or *in vivo*, leaving the impact of mechanical or electromagnetic intervention on this pathway in the nervous system unclear.

In summary, we have consolidated the findings on intracellular pathways modulated by various mechanical and electromagnetic stimulation (Table 1). These cellular pathways are highly interconnected, though the extent of crosstalk between pathways is context-dependent (Figure 1). Generally, PKC activates Raf and MEK, which are upstream of the MAPK signaling pathway (Aksamitiene et al., 2012). Conversely, activation of Akt signaling provides negative feedback to the Raf–ERK pathway (Mendoza et al., 2011), while inhibition of Akt in neurons enhances and promotes JNK activity (Hollville et al., 2019). Akt activation also phosphorylates YAP, leading to its sequestration in the cytoplasm (Basu et al., 2003). Meanwhile, Rho signaling is implicated in the mechanically induced activation of YAP/TAZ (Dupont, 2016). It also inhibits Akt while promoting p38 MAPKs and JNK in neuronal cells (Stankiewicz and Linseman, 2014; Moya and Halder, 2019). Consistent with these interactions, YAP/TAZ activation further enhances JNK cascades (Wang et al., 2016). Moreover, studies have shown that mechanical force-induced integrin-Src-FAK activation triggers multiple related pathways, including MAPK, PI3K-Akt, YAP/TAZ, and Rho-ROCK signaling (Moya and Halder, 2019; Schwartz, 2010).

### Mechanoreceptors in neuronal and glial cells

#### Cytoskeletal components and adhesion molecules

Currently, it is understood that cells sense mechanical axial and shear stress through integrin-actomyosin dynamics (Schwartz, 2010). Integrins, which bind to ECM proteins like fibronectin or laminin, connect to the cytoskeleton-like actin when forces are applied. These mechanical cues trigger changes in integrins, initiating the formation of linkages with actin and other focal adhesion complex molecules, including Src, focal adhesion kinase (FAK), and proline-rich tyrosine kinase 2 (PYK2). Subsequent mechanical stretching of cells or use of rigid extracellular materials activates of various signaling cascades, including MAPK, Rho GTPases, PI3K, and increased cytoplasmic calcium levels (Schwartz, 2010). These cascades, in turn, further regulate cytoskeletal dynamics. In particular, YAP/TAZ signaling is influenced by ECM-integrin interactions with cytoskeletal F-actin, which promotes TEAD transcription factor activation (Panciera et al., 2017). This integrin-FAK-mediated response has also been implicated in ultrasound-mediated cellular responses (Tang et al., 2006).

Neurons and glial cells express distinct integrin subtypes depending on the brain regions and cell type (Pinkstaff et al., 1999). Notably, integrin *α*3β1 plays a crucial role in synaptic plasticity, while integrin αv regulates the migratory functions of glial cells (Wang et al., 2005; Lilja and Ivaska, 2018; Milner et al., 1996). In hippocampal neurons, the activation of integrin-dependent receptor-like protein tyrosine phosphatase α (RPTPα) has been implicated in mechanical sensory signaling (Kostic et al., 2007). In addition, PYK2 expression, along with FAK, are highly enriched in CNS neurons compared to other organ tissues and is involved in synapse and neurite formation (Menegon et al., 1999; de Pins et al., 2021). Although mature neurons typically do not express YAP and show low levels of integrin expression post-development, mechanical stretching might have a more limited impact on neurons than on other glial cells. Nonetheless, promoting stretch signaling appropriately could support axonal regeneration and plasticity, as overexpression or the activation of integrins has been shown to enhance neurite outgrowth in adult neurons (Condic, 2001; Ivins et al., 2000).

#### Activation of cellular membrane mechanosensory receptors and mechanical stress-gated channels

Members of the transient receptor potential (TRP) ion channel family serve as key sensors of mechanical stimulation in the nervous system. These channels are broadly expressed in the brain, spinal cord, and DRG neurons (Pedersen et al., 2005) and play a crucial role in mechanosensation, responding to a wide range of mechanical stimuli (Pedersen et al., 2005). Among them, the TRP Vanilloid receptor family (TRPV), which consists of Ca^2+^-permeable ion channels, is particularly significant for its activation by mechanical stress in the mammalian nervous system. For instance, TRPV2 is activated by cell stretching in neurons (Shibasaki et al., 2010), while TRPV4 in astrocytes responds to various mechanical stresses, including osmotic pressure (Kanju and Liedtke, 2016; Benfenati et al., 2007). Other members of the TRP family, such as TRPC, TRPM, and TRPA1, are also highly expressed in the mammalian CNS (Sawamura et al., 2017; Kheradpezhouh et al., 2017). Although the expression of these channels is abundant in neurons and glial cells, their activation in response to mechanical stress has not been well-characterized. Recently, TRPA1 has been highlighted for the role it serves in sensing fluid shear stress and regulating calcium influx in *Drosophila* enteroendocrine cells (Gong et al., 2023).

Purinergic P2 receptors also play a role in mechanosensation. The P2X and P2Y receptor subtypes are widely expressed in the brain and spinal cord, including in neurons and glial cells (Franke et al., 2006). Among them, the G protein-coupled P2Y_2_ receptor contains an integrin-binding site that mediates cytoskeletal signaling and stimulates PKC activation (Peterson et al., 2010). Furthermore, the P2Y_2_ receptor is particularly implicated in nerve injury and the pathology of neurodegenerative diseases such as Alzheimer’s disease (Peterson et al., 2010). In terms of mechanical stress, the P2Y_2_ receptor is activated by fluid shear stress, initiating intracellular cascades in endothelial cells (Sathanoori et al., 2017; Wang et al., 2015). In rat astrocytes, research indicates that stretch-induced Akt phosphorylation is mediated by P2 receptors (Neary et al., 2005). While P2 receptors appear to function as mechanosensors for various cell types, their specific roles in neurons, particularly in the context of mechanical or electromagnetic stimulation, remain unclear.

Piezo 1 and 2 are among of the most studied mechanically activated ion channels over the past decade. Initially discovered in the neuronal cell line Neuro2A and DRG cells, these channels play crucial roles in mechanotransduction (Coste et al., 2010). In the nervous system of mammals, Piezo1 is broadly expressed, whereas the expression of Piezo2 is more selectively found in sensory neurons, cortical and hippocampal pyramidal neurons, cerebellar Purkinje cells, and olfactory bulb neurons (Zong et al., 2023; Wang and Hamill, 2021). Piezo channels are now recognized as key mechanosensors that transduce signals by opening cation channels in response to mechanical stimuli including stretch, fluid shear stress, and ultrasound stimulation.

Recently, the role of Piezo channels in neurophysiology and related pathologies has garnered increasing attention, leading to several comprehensive reviews on their significance in the nervous system (Zong et al., 2023; Zheng et al., 2023; Fang et al., 2021). While Piezo1 primarily mediates cation influx in response to mechanical stimuli, its activation through classical cellular pathways, rather than mechanical sensation, yields varying outcomes. For example, activation of Piezo1 has been shown to reduce axonal outgrowth in mammalian neurons (Song et al., 2019), whereas, pharmacological activation of Piezo1 has been linked to demyelination in the mouse brain (Velasco-Estevez et al., 2020). These findings underscore the need for further research to elucidate the specific conditions required to trigger classical intracellular pathways and how these pathways may differ compared to other mechanosensors.

Several types of G protein-coupled receptors (GPCRs), including angiotensin II type 1 receptors and adhesion GPCRs (aGPCRs), have also been recognized for their mechanosensitive properties (Wilde et al., 2022). In particular, aGPCRs are known to be responsive to various mechanical stimuli including, shear stress, pressure, and stretching. These are hypothesized to regulate processes in the nervous system involved in development, synapse formation, and myelination (Langenhan et al., 2016). Various subfamilies of aGPCRs are widely expressed in the mammalian CNS, with their expression patterns varying by region and developmental stage (Ehrlich et al., 2018). Among them, GPR 56 and 68, which are expressed in both mouse and human brains, have been shown to be activated in response to mechanical stretching and fluid shear stress *in vitro* (Wei et al., 2018; Yeung et al., 2020; Ozkan et al., 2021). Activation of GPR56 is known to promote PKC activation and the Rho-ROCK signaling pathway (Ganesh et al., 2020). These findings suggest that other aGPCRs expressed in neurons and glial cells may also respond to mechanical forces, highlighting their potential mechanosensitive role and opening a new avenue for further investigation.

Studies suggest cells can sense electromagnetic stimulation through direct activation of voltage-gated calcium channels, resulting in an influx of calcium ions (Pall, 2013; Grassi et al., 2004). It is hypothesized that ion oscillations induced by electromagnetic stimulation disrupt the electrochemical balance of cell membranes (Panagopoulos et al., 2002). However, the specific types of calcium channels involved in this response have not been clearly elucidated. Another possibility is that thermosensitive TRPV channels are activated in response to the heat generated during electromagnetic stimulation (Stanley and Friedman, 2019). Additionally, given the abundant expression of TRPV channels in the brain and spinal cord, TRPV-mediated calcium influx may also serve as another plausible mechanism for cellular signaling in response to electromagnetic stimuli in the nervous system (Gunthorpe et al., 2002). In summary, the key mechanosensors discussed are succinctly illustrated in Figure 1.

## Discussion

This review explores the key features of mechanically activated cellular pathways and related mechanosensors, with a particular focus on the mammalian nervous system. The studies discussed here primarily focused on two types of stimulation: mechanical stretching and electromagnetic stimulation. Electromagnetic stimulation has recently gained attention, particularly with the U.S. FDA’s approval of repetitive TMS in 2008 for treating major depressive disorder, and the approval of pulsed EMF for musculoskeletal disorders (George et al., 2013; Waldorff et al., 2017). Beyond TMS, there has been a growing interest in the use of ultrasound stimulation to modulate the nervous system, particularly in the context of mental health (Hameroff et al., 2013). The immense potential for practical applications of these techniques continues to attract significant interest in the field. While these methods can activate substantial cellular signaling pathways, the specific mechanosensitive channels or receptors remain largely undefined. As electromagnetic and ultrasound stimulation continues to expand in therapies and rehabilitations for neurodegenerative disorders and injuries, further elucidation of related mechanisms on neural cells is highly anticipated.

Among the cellular pathways responsive to mechanical and electromagnetic stimulation, the MAPK and Akt pathways have been extensively studied. Akt activation is commonly reported across various types of stimuli with the exception of high-frequency electromagnetic stimulation. However, the activation of specific subgroups of MAPKs may depend on the type of stimuli and cells involved. In neurons, phosphorylation of ERK and JNK is commonly triggered by various stimuli *in vitro*, while p38 MAPKs were not. In contrast, *in vivo* studies using compression models have shown upregulated ERK and p38 MAPK. However, these results should be cautiously interpreted as *in vivo* application using compression may cause cellular damage and inflammatory responses that confound the effects of mechanical stimulation.

Microglia exhibited different responses to ultrasound and electromagnetic stimuli compared to other cell types. Reports on the YAP/TAZ pathway have primarily focused on oligodendrocytes and Schwann cells, particularly in relation to changes in their myelination functions. In contrast, reports on the Rho-ROCK pathway have largely focused on matrix stiffness-mediated cellular differentiation in neural stem cells (Kang et al., 2020; Oyama et al., 2021).

A significant limitation of the current methodology lies in the difficulty of determining the appropriate range of force or intensity thresholds required for different types of mechanical stressors. While this is relatively straightforward in the case of using low-frequency electromagnetic stimulation (e.g., ~50 Hz) or high GHz microwave exposure, the precise magnitude of stimulation needed to achieve beneficial effects or avoid pathophysiological consequences remains an area requiring careful consideration. This issue necessitates thorough investigation across various contexts to accurately assess the cellular-level impacts that could lead to either positive outcomes or adverse effects. Additionally, distinguishing between calcium influx due to inflammatory reactions (e.g., injury) and that induced by mechanical stimuli *in vivo* is challenging. For example, Akt and p38 MAPK activation observed in *in vivo* models may be associated with subsequent neuroinflammation (Asih et al., 2020; Pu et al., 2023). Thus, we have prioritized *in vitro* studies to interpret results, though it is crucial to acknowledge *in vivo* results. Translating promising *in vitro* findings into *in vivo* contexts is necessary for identifying precise mechanisms. Micro-level techniques, such as AFM-based compression or microfluidic chips, offer the potential for more precise *in vitro* experimentation on mammalian neurons and glial cells (Onal et al., 2022). These methods could enable more detailed investigations into the activation and deactivation of specific cell signaling pathways.

Tissue stiffness-mediated mechanical stress was not explicitly addressed in this review because stretching and mechanical compression methods offer similar forms of mechanical stress. Comprehensive reviews done on tissue stiffness sensing, and related cellular pathways are available for further reading (Pillai and Franze, 2024; Motz et al., 2021; Schwartz, 2010; Janmey et al., 2020).While we did not discuss all types of mechanical stimuli and their molecular, it is important to emphasize that understanding stress type-specific molecular pathways is critical for comprehending clinically relevant biological responses in the CNS. This consideration is essential even for models not directly related to the traumatic CNS injury field.

In conclusion, various mechanical and electromagnetic stimuli can activate significant cellular signaling pathways in different ways, leading to diverse outcomes in the nervous system. Further research is needed to deepen our understanding of the precise biomolecular mechanisms underlying the therapeutic effects of mechanical stimulation on neuroregeneration and neurorehabilitation (Huie et al., 2017).
